# A case for qualitatively driven mixed methods in nursing research: a methodological discussion

**DOI:** 10.1177/17449871261421123

**Published:** 2026-04-18

**Authors:** Jennifer Hamilton, Simon Cooper, Irene Ngune, Amanda Cole, Richard Bostwick, Debbie Louise Massey

**Affiliations:** Senior Lecturer, School of Nursing and Midwifery, Edith Cowan University, Australia; Professor, School of Nursing and Midwifery, Edith Cowan University, Australia; Associate Professor, School of Nursing and Midwifery, Edith Cowan University, Australia; Senior Lecturer, School of Nursing and Midwifery, Edith Cowan University, Australia; Associate Professor, School of Nursing, Curtin University, Australia; Professor, School of Nursing and Midwifery, Edith Cowan University, Australia

**Keywords:** mental health, methods, philosophy, research design, research, phenomenology

## Abstract

Mixed methodology continues to grow as a rigorous and respected research design. However, the value of qualitatively driven mixed methods research within nursing is underrepresented. Qualitatively driven mixed methods, where qualitative data dominate meaning-making, have the potential to capture critical experiential elements pertinent to nursing research while supporting empirical rigour. This methodology paper argues for the importance of qualitatively driven mixed methods approaches within nursing research to enable greater understanding of complex phenomenon. Through discussion and case example the role of qualitatively driven mixed methods, including within a sequential explanatory design, are presented. Nurses and midwives are well placed to design and conduct health research, and this paper provides methodological guidance and advances epistemological understanding of qualitatively driven mixed methods within research to improve staff and patient outcomes. For health and social care policy, these methods provide rich contextual data that can inform policy development and evidence-based practice. This methodological discussion piece highlights how qualitatively driven mixed methods enable greater understanding of complex phenomenon and experiential insights.

## Introduction

The central premise of mixed methods research (MMR) is that through the combination of quantitative and qualitative approaches, the research problem can be more effectively explored and understood (Creswell and Clark, 2018). In recent years, MMR has continued to evolve as a rigorous and respected research design (Smajic et al., 2022). This approach provides integration of quantitative and qualitative data sources, validation of findings, flexible approaches and improved identification of relationships or confounding factors. Together these enable researchers to address complex problems comprehensively and identify meaningful insights through generation of metainferences (Barton, 2020; Mayoh & Onwuegbuzie, 2015; Scammon et al., 2013; Younas et al., 2025). Such insights are crucial when developing informed and effective interventions with real-world impact. Consequently, high-quality, rigorous MMR compliments traditional methodologies and offers more thorough exploration of a research question and new ways of knowing. Despite the strengths of MMR, Gilbert (2006) acknowledged the tension experienced by researchers as they navigate complex funding structures for healthcare research that reinforces and privileges quantitative approaches. Gilbert (2006) and Doyle et al. (2016) challenged this worldview arguing that mixed methods researchers complete, triangulate, and expand the contribution of the single research approach. Within health research, mixed methods with qualitative dominance offer a unique opportunity to capture both quantifiable patterns and rich experiential insights. This dual approach is invaluable when exploring complex and nuanced aspects of human experience and health phenomena.

However, although MMR has gained increasing acceptance, it remains underdeveloped within nursing and midwifery (Wasti et al., 2022), partly due to challenges in education and understanding regarding its practical application as a methodology – including data integration and maintaining consistency with conceptual and philosophical frameworks, which can be challenging for researchers (Crawford, 2019). Guidance to assist researchers in establishing design methodologies within mixed methods studies, which maintain philosophical consistency, are needed to enhance quality and rigour of research output (Harrison et al., 2020).

This paper addresses this gap by providing an exemplar of a qualitatively driven explanatory sequential mixed methods study, demonstrating practical approaches to data integration while maintaining philosophical coherence between pragmatism and phenomenology. A sequential explanatory design study has been selected as this approach is often presumed to be quantitatively dominant and driven, due to an initial – and often larger – quantitative data collection stage. However, the nuance of data dominance and drive within mixed methods studies provides opportunity to consider how world view impacts: study design, measurement tool selection, integration, and interpretation of data.

## Background

### Qualitative dominance versus qualitative drive

Oftentimes mixed methods studies across disciplines hold a greater emphasis on quantitative data (Morgan and Hofffman, 2021). However, *qualitative dominance*, often understood where qualitative data are collected first or has a larger representation (e.g. QUAL > quant), may be best suited when a research question requires in-depth exploration of specific experiences to facilitate a comprehensive understanding of the problem (Mayoh & Onwuegbuzie, 2015). When studying health workforce issues, patient care interventions or experiences, a greater emphasis on qualitative data may be particularly valuable as it allows researchers to capture the nuanced realties of lived experience while maintaining empirical foundations (Mason, 2006). Moreso, studies which have a preceding or larger volume of quantitative data can remain qualitatively *driven*, whereby qualitative elements provide, direction, clarification or illumination of the research problem, or issue, and real-life experience or application for practice. This rationale for qualitatively driven research stems from the complexity of human experiences within healthcare (Demkowicz et al., 2025).

### The role of MMR in nursing

The dual influence of art and science in nursing practice reflects the unique opportunity to address both bioscience and human experience (Salum et al., 2020). Research suggests neither quantitative nor qualitative approaches alone may fully capture the complexity of healthcare (Demkowicz et al., 2025; Palinkas et al., 2011a). This duality highlights the need for research methods which capture both measurable outcomes and the richness of human experience. MMR is particularly well suited to interdisciplinary fields, as it integrates quantitative precision with qualitative depth (Creswell and Plano Clark, 2018), while also enhancing credibility, generalisability and contextualisation, allowing for more meaningful conclusions (Irvine et al., 2020). A core principle of MMR lies in the deliberate integration of both quantitative and qualitative data within a single study to gain a more comprehensive understanding of the research problem (Creswell & Clark, 2018). MMR emphasises the integration of both methodologies across all design stages to generate deeper insights. Although contemporary research methods continue to evolve, including increasing representation of co-design and participatory action models, most scholarly sources advising on mixed methodologies identify the following three main design methods (Fàbregues et al., 2020):

(1) Convergent design: The researcher collects and analysis quantitative and qualitative data independently prior to merging and interpreting the data.
(2) Exploratory sequential design: Qualitative data are collected followed by quantitative data. Interpretation includes how the quantitative data provide further insights.
(3) Explanatory sequential design: Quantitative data are collected, followed by qualitative data. Interpretation includes how qualitative data explain initial quantitative results.

Although the steps of MMR – data collection, analysis and interpretation – appear straightforward, the reality is more complex. Key challenges include justifying the approach (Irvine et al., 2020; Oliveira, 2021), achieving consistent integration across all stages of the project (Younas, et al., 2019), and addressing critical questions such as: Are the research questions formulated for a mixed methods design? Do quantitative and qualitative data collection methods complement each other? Does the research team have the expertise to effectively analyse, integrate, and interpret both data types? Has a philosophical framework been thoughtfully applied throughout the project? To date, there have been limited texts providing an overview of MMR for those considering its use in healthcare research and specifically within nursing. The focus instead has been on specific issues within mixed methods, including the paradigm wars and the issue of data integration (Demkowicz et al., 2025; Doyle et al., 2016; Palinkas et al., 2011a).

Addressing these challenges requires expertise in quantitative, qualitative, and mixed methodologies – as well as careful consideration from conception to dissemination, thus ensuring methodological rigour enhances the validity of MMR and captures the complexity of nursing practice. When addressing these aspects, MMR provides a rich and nuanced understanding of research problems by offering multiple ways of examining research questions – capturing both ‘what’ is happening through quantitative measures and understanding ‘why’ or ‘how’ through qualitative approaches (Wasti et al., 2022). This combination creates a holistic picture and bridges the art and science of nursing care, offering solutions with meaningful real-world impact that neither approach could achieve independently (Wasti et al., 2022).

Combining data sources, validating findings across methods, and uncovering nuanced relationships provides flexibility and promotes rigour within MMR thus providing the opportunity to establish a comprehensive understanding of complex phenomena and connecting objective data with subjective insights (Creswell and Plano Clark, 2018). These factors are necessary when addressing complex research problems to identify meaningful implications (Barton, 2020; Mayoh and Onwuegbuzie, 2015) – both of which are pertinent when addressing health-related issues.

With the above issues in mind, researchers often face methodological questions which extend beyond available guidelines and reporting standards, such as the APA *Journal Article Reporting Standards for Mixed Methods Research* (*JARS-Mixed*; APA, 2024). Although these standards provide general guidance on reporting requirements for mixed methods studies, they offer limited practical direction, specifically when implementing qualitatively driven sequential designs. This paper responds to these methodological questions by offering an exemplar illustrating key considerations when designing and conducting qualitatively driven MMR, with a focus on methodological rigour and integration.

### Philosophical considerations when applying qualitatively driven MMR

Although phenomenology and pragmatism are often identified as independent traditions, their alignment and merging has been recognised in literature (Bourgeois, 2002; Hackett, 2018; Ramanadhan et al., 2021). In this merging of phenomenology and pragmatism, the content of experience can identifiably influence events to which the individual and others have been exposed (Bourgeois, 2002). By incorporating both philosophical paradigms within MMR, researchers can uncover subjective truth of perception and knowledge drawn from real-world experience. This combination positions research findings to be relevant and actionable with real-world application for both clinicians and consumers in everyday practice.

Such a philosophical approach may assist researchers in developing qualitatively driven design within explanatory sequential studies. Researchers often struggle to achieve true qualitative emphasise within this method, as it is frequently presumed this design must be quantitatively driven. Reviews of MMR in mental health and nursing consistently demonstrate that quantitative methods remain dominant. Palinkas et al. (2011b) found that quantitative methods dominated 74% of mixed methods studies within their review, whereas Irvine et al. (2020) similarly reported three-quarters of published studies prioritised quantitative approaches. Badu et al. (2019) further confirmed this trend in their integrative review, while noting mixed methods approaches were becoming increasingly common, most studies employed qualitative components primarily to explain or elaborate on quantitative findings. This highlighting, the limited number of qualitatively dominant explanatory sequential studies, where typically the quantitative phase is used merely to identify purposeful samples for qualitative interview stage, rather than sophisticated integration (Morgan and Hoffman, 2021). Although in most explanatory sequential studies identified, qualitative data are limited to explain quantitative findings (Morgan and Hoffman, 2021). However, within qualitatively driven explanatory sequential studies, initial quantitative data provide orientation and focus, whereas qualitative methods allow deeper exploration and explanation for meaning-making. This dialectic approach creates a space for both deductive and inductive reasoning, transcending the so-called quantitative–qualitative divide and providing a wider explanatory lens (Mason, 2006).

## A case example of maintaining qualitative drive within sequential an explanatory design

The following exemplar is informed by a parent study which adopted a qualitatively driven sequential mixed methods explanatory design (Schoonenboom and Johnson, 2017). This design has been selected to provide awareness that ‘driven’ does not equate to the volume or sequencing of data types, rather the lens applied to the study design and interpretation in meaning-making. This study explored the impact of an innovative approach to staff clinical supervision within mental health nursing, which integrated evidence-based principles with structured reflective practice. This approach was specifically designed to enhance clinical decision-making and promote alternative interventions to reduce restrictive practices.

The study aimed to determine how the intervention affected both clinical supervision and practice within an inpatient mental health setting. And answer the research question, how does the clinical supervision approach affect clinical supervision and evidence-based practice implementation? Ethical approval was obtained from Edith Cowan University and Ramsay Healthcare Human Research Ethics Committees. All participants volunteered to partake in the study and provided informed consent.

Pragmatism guided the study, ensuring that knowledge was grounded in the context of contemporary clinical practice (Dolan et al., 2022), acknowledging that meaningful knowledge emerges through solving real-world problems (Prasad, 2021), whereas the philosophical underpinning of phenomenology captured participants (care givers) lived experiences and perspectives and was fundamental to the study design (Dodgson, 2023; Hamilton et al., 2023).

**Figure 1.**
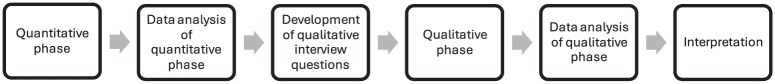
Adaptation of explanatory sequential designs. Note this figure was adapted from Creswell and Plano Clark (2018: 853).

The explanatory sequential design, which involves initial quantitative data collection followed by qualitative data collection, is depicted in Figure 1 and provided structure to the study through six phases, ensuring a cohesive approach to data integration.

### Data sources in the exemplar study

In keeping with the explanatory sequential design approach quantitative data collection via surveys (*n* = 67) were conducted first, followed by qualitative interviews (*n* = 8) to further explore the significant results from the initial phase (Doyle et al., 2016). The interview sample population sufficiently met data saturation, as aligned with Ritchie et al. (2014) whereby the interviewer used probing questions until they believed that a full understanding of participants perspectives had been collected. The qualitative interviews were analysed using Interpretive Phenomenological Analysis (IPA) (Smith et al., 2009), which allowed for in-depth exploration of participants’ lived experiences and the meaning they attributed to clinical supervision and their clinical practice (Smith et al., 2009).

Quantitative data were collected using the Manchester Clinical Supervision Scale-26^©^, a self-report questionnaire administered before and 9 months after the introduction of the intervention. The survey was selected because it aligns with capturing participant perspectives, measuring the effectiveness of clinical supervision as perceived by the participants themselves. This survey provided alignment with the phenomenological underpinnings through perception of participant experiences. Descriptive and inferential analysis informed development of an interview guide (Ivankova et al., 2006; Liem, 2018), whereby the quantitative results portraying participant experiences and perceptions could be further explored. Interviews then explained quantitative findings and addressed unanswered research questions (Draucker et al., 2020; Liem, 2018), maintaining the explanatory sequential design (Creswell and Plano Clark, 2018). Connection between quantitative and qualitative phases was reinforced by purposeful nested sampling, where participants who completed the quantitative phase were invited to partake in the qualitative phase (Ivankova et al., 2006).

### Exemplar study key findings

As this is a discussion paper, detailed findings from the exemplar study are not presented. Instead, an overview of the key results and integrated findings are presented to illustrate the approach.

#### Quantitative results

Nursing staff satisfaction with clinical supervision improved following the intervention as measured by the MCSS-26^©^ total scores (pre-intervention 69.54, *SD* 16.06; post-intervention 71.47, *SD* 13.98). The survey also identified improvement in nursing staff’s perception of the restorative and formative domains of clinical supervision. The restorative domain mean score increased from 28.43 (*SD* 5.99) pre-intervention to 29.29 (*SD* 3.95) post-intervention. The formative mean score increased from 20.10 (SD 5.62) pre-intervention to 20.63 (*SD* 13.98) post-intervention. The normative domain demonstrated the lowest scores among the three domains, with mean score decrease from 21.08 (SD 5.98) pre-intervention to 20.76 (SD 6.33) post-intervention, with particular challenges identified in the ‘finding time’ subscale.

#### Qualitative results

The qualitative findings further explained the satisfaction levels and the changes seen in perception domains. Interpretive phenomenological analysis identified four themes (i) improved therapeutic relationships and patient centred care, (ii) improved staff communication, and teamwork, (iii) barriers to clinical supervision engagement, and (iv) assistance with the change process.

#### Integrated findings

The findings demonstrate that the intervention had a positive impact on nursing staff’s perception of both clinical supervision and evidence-based practice implementation. The modest improvement in overall survey scores aligned with interview data reporting positive attitudes towards the intervention. Both datasets identified time-constraints as a significant challenge, which often limited participants engagement with the intervention during the study period. Overall, the integrated findings suggest the intervention effectively supported nursing staff through the change process while improving satisfaction with clinical supervision, despite ongoing challenges with logistical barriers and workplace demands.

### Details of data integration within exemplar study

Integration of data was critical within the study and achieved at various stages. A building approach was used whereby quantitative data informed development of the interview guide for qualitative data collection (Draucker et al., 2020). Identifying key areas from quantitative data which required further exploration, interview guides were designed to mirror specific survey questions; allowing richer, narrative-based explanations within qualitative phase (Creswell and Plano Clark, 2018). This was conducted through identifying the initial insights gained through quantitative data analysis to directly inform the development of interview questions. For example, after identifying the normative domain consistently received the lowest mean scores in the surveys, specific interview questions were designed to explore barriers to attendance and motivations for participation, (e.g. ‘Do you find it difficult to get off the ward to attend clinical supervision?’). Several interview questions intentionally mirrored survey items which required further clarification, (e.g. ‘Do you find clinical supervision takes you away from your “real work”?’). Similarly, interview questions were crafted to explore evidence-based practice implementation and aspects of the research question which the survey failed to address, such as integration of the evidence-based practice principles, (e.g. ‘How does clinical supervision impact your personal practice?’). Connection between quantitative and qualitative phases were further reinforced by purposeful sampling, whereby participants who completed the quantitative phase were invited to partake in qualitative phase (Ivankova et al., 2006).

**Figure 2.**
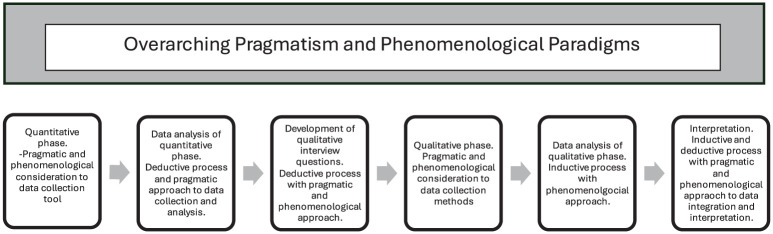
Adaptation of explanatory sequential design with overarching pragmatic and phenomenological paradigm.

Data integration extended beyond analysis and reporting. Initially, the quantitative survey data and qualitative interview data were analysed independently to maintain methodological integrity. Descriptive and inferential analyses were conducted on the quantitative dataset. The quantitative results were then ‘qualitised’ and presented in a narrative format. Subsequently, the ‘qualitised’ data were merged with the interview data to enable comparative analysis and synthesis (Castro et al., 2010). This merging process specifically combined quantitative results on effectiveness perceptions with qualitative narratives of supervision and practice experiences, offering complementary insights that illuminated how participants experienced and perceived the intervention and gain a better understanding of the complex research problem in the parent study (Scammon et al., 2013). Integrated findings were presented as narrative discussion, whereby quantitative and qualitative findings were synthesised into a cohesive narrative, enabling increased contextualisation (Fetters et al., 2013). Through this process, metainferences were developed providing an integrated interpretation of findings which informed implications and recommendations for practice, policy, and research (Younas et al., 2025). The design stages including philosophical alignment are further illustrated in Figure 2.

The integration of methods across various stages exemplifies the importance of thoughtful consideration in MMR (Creswell and Plano Clark, 2018; Fetters et al., 2013; Younas et al., 2019). Embedding both quantitative and qualitative approaches within design, analysis, and reporting phases achieves a robust exploration of research questions and integration. This integration illuminates both knowledge and truth of experience by bringing together measurable outcomes with subjective lived experience. Specifically, quantitative data provide empirical knowledge through measurable indicators, whereas qualitative data capture the subjective truth derived from participant’s lived experiences of the phenomenon. Philosophical underpinnings allowed for exploration of individual perspectives, providing rich descriptions of how participants make sense of the intervention within their specific socio-cultural contexts (Eberle and Schnettler, 2019). During integration, quantitative findings were contextualised by participants’ narratives, creating a more comprehensive understanding that neither approach could achieve independently. This process offered deeper insights into how participants experienced and perceived the intervention, illuminating the phenomenon of interest through both objective measurements and subjective meaning-making (Scammon et al., 2013). The truth reflected in this integration represents the collective presentation of meaning derived by participants’ experiences and perceptions, acknowledging that knowledge is contextually situated within the specific realities of the study setting (Barton, 2020; Sheets-Johnstone, 2020).

## Discussion

The exemplar presented in this paper demonstrates the feasibility and value of qualitative dominance within an explanatory sequential mixed methods design. This approach aligns with the broader literature whereby qualitative methods provide depth of understanding, whereas quantitative methods test hypotheses providing breadth of understanding. Our exemplar extends this by illustrating how qualitatively driven explanatory sequential designs can be effectively implemented to explore complex phenomena.

The prevalence of quantitatively driven MMR documented by Palinkas et al. (2011b) and Irvine et al. (2020) represents more than a numerical imbalance – it reflects deeper methodological assumptions about the relationship between empirical data and experiential knowledge. Our exemplar challenges these assumptions by demonstrating how explanatory sequential designs can effectively prioritise qualitative inquiry without compromising on methodological coherence. Thus, addressing a key challenge in MMR – maintaining philosophical consistently while integrating differing datasets (Harrison et al., 2020).

Mason’s (2006) dialectic approach advocates for transcending the quantitative–qualitative divide through strategic integration. Arguing that mixed methods can create a dialogic relationship between different ways of knowing and seeing. Such conceptual scaffolding aligns with Morgan’s (2014) recommendation that a pragmatic approach adopts a process of inquiry where knowledge is based on experience and methodological choices should be guided by the context and purpose of their adoption. The resultant dialogue between quantitative and qualitative principles creates what Mayoh and Onwuegbuzie (2015) described as mixed methods phenomenological research, where phenomenological inquiry is enhanced rather than compromised by integration with quantitative methods.

A key contribution of our approach is the deliberate prioritisation of qualitative inquiry, despite beginning with quantitative data collection – a sequence often assumed to necessitate quantitative dominance. This reversal from the more typical adoption demonstrates how researchers can leverage the strengths of methodologies while maintaining philosophical coherence (Ivankova et al., 2006). Using quantitative data to inform and focus subsequent qualitative exploration, rather than as the primary basis of evidence, the exemplar study achieves a nuanced understanding of participants’ experiences with the clinical supervision intervention.

Data integration, a key aspect of MMR, can be thoughtfully implemented through all design stages (Draucker et al., 2020; Fetters et al., 2013). Although Palinkas et al. (2011a) identified three approaches to data integration – merging, connecting, and embedding – our exemplar provides a practical illustration of how connecting begins in the design planning stage. This involves carefully designed research questions and selection of quantitative data collection tools, which align with phenomenological and qualitative paradigms centred on participant perception. Strategically developed interview questions that mirror specific survey items exemplifies how researchers can connect datasets while maintaining methodological integrity. This process enhances rigour through thoughtful integration across all stages of the research process, not just during analysis (Harrison et al., 2020).

Bringing together phenomenology and pragmatism, responds to what Palinkas et al. (2011a) identified as primary reasons for implementation research: to examine both the content and context of an intervention. Our approach pragmatically asks ‘what works’ while phenomenologically considering stakeholder perspectives and lived experiences. This dual philosophical framework enables qualitative dominance to transcend mere data integration and achieve genuine epistemological integration – an advancement over existing methodological guidance.

Reporting standards such as the APA *Journal Article Reporting Standards for Mixed Methods Research* (*JARS-Mixed*; APA, 2024) provide general guidance on reporting mixed methods studies, including the need to describe philosophical approaches and data integration procedures. However, they offer limited guidance on how to achieve philosophical coherence between paradigms or how to implement integration techniques at a practical level. Our exemplar addresses this gap by providing nursing and healthcare researchers with actionable guidance that goes beyond reporting requirements to support methodological integrity within a qualitatively driven approach.

The qualitatively driven approach presented within this paper is particularly valuable within mental health nursing research, where understanding lived experience and subjective perceptions is central to developing effective interventions (Demkowicz et al., 2025). The exemplar study demonstrated how initial quantitative findings (showing modest improvements in supervision satisfaction scores) gained deeper meaning when contextualised through qualitative inquiry (revealing how the intervention improved therapeutic relationships and team communication, and supported change processes despite ongoing challenges). This integration illuminated both knowledge and truth of experience by bringing together measurable outcomes with subjective lived experience.

Researchers using MMR in nursing position their contribution to knowledge politically as well as empirically and clinically because MMR transcends clinical practice, patient advocacy, and institutional systems of power by embracing typology of knowledge which includes practice, technical and political (Gilbert, 2006). We argue that by integrating and valuing qualitative and quantitative approaches methodological pragmatism is embraced, but importantly political choices on types of knowledge valued in shaping healthcare are illuminated, challenged, and embraced. However, the paradigms tension previously discussed underscores the political nature of mixed methods: it can either reinforce dominant clinical norms or serve as a tool for critical inquiry and transformation in nursing practice (Gilbert, 2006).

## Considerations on adopting this approach

Adopting qualitatively driven MMR provides opportunity for a robust and nuanced exploration of research problems. However, qualitatively driven explanatory sequential designs necessitate considerable time and resources, potentially constraining implementation in certain study environments. Integration requires the research team hold skills in both quantitative and qualitative approaches – expertise that many teams may struggle to combine effectively in practice (Harrison et al., 2020). Furthermore, achieving authentic philosophical cohesion between pragmatism and phenomenology may present as a challenge, which researchers must navigate with methodological reflexivity. These constraints highlight considerations for valuable implementation of this approach.

## Conclusion

Although literature provides various applications of mixed methods in implementation research, detailed exemplars of qualitatively driven explanatory sequential designs are underrepresented. This paper addresses this gap by providing a transparent account of methodological decision-making, philosophical integration and analytical procedures which prioritise qualitative inquiry while maintaining the benefits of initial quantitative inquiry. This exemplar illustrates how sequential design can maintain qualitative priority despite quantitative initiation, providing a resource for nurses and midwives when conducting research which promotes development of new knowledge.

**Key points for policy, practice and/or research**

- Qualitatively driven mixed methods, where qualitative data dominate meaning-making, do not require qualitative data to be collected first.
- Qualitatively driven mixed methods provide a robust approach which may address complex health phenomena.
- Methodological guidance to assist nurses and midwives, who are well placed to design and conduct research, assists with research process, capability and quality to improve staff and patient outcomes.
- MMR in nursing offers political value by challenging which types of knowledge are privileged, illuminating power dynamics in evidence bases.
- Methodological guidelines enhance research quality and capabilities, potentially transforming practice and clinical outcomes through improved evidence-based practice approaches, leading to equitable and effective mental healthcare service design and policy development.
